# Comprehensive cardiac magnetic resonance imaging and spectroscopy reveals a high burden of myocardial disease in HIV infection

**DOI:** 10.1186/1532-429X-15-S1-O25

**Published:** 2013-01-30

**Authors:** Cameron Holloway, Ntobeko Ntusi, Joseph Suttie, Masliza Mahmod, Emma Wainwright, Genevieve Clutton, Gemma Hancock, Philip Beak, Abdelouahid Tajar, Stefan K Piechnik, Jurgen E Schneider, Kieran Clarke, Lucy Dorrell, Stefan Neubauer

**Affiliations:** 1 University of Oxford, Oxford, UK

## Background

Human immunodeficiency virus (HIV) infection continues to be endemic worldwide. Whilst treatments are successful, it remains controversial whether patients receiving optimal therapy for HIV infection have structural, functional or biochemical cardiac abnormalities which may underlie the increased cardiac morbidity and mortality. Our main objective was to characterise myocardial abnormalities in a contemporary group of HIV-infected individuals.

## Methods

This was a cross-sectional observational study of patients with HIV infection. One hundred and forty three volunteers were recruited in this study, among those 104 were asymptomatic HIV-infected subjects aged ≥ 18 years (91 receiving cART) without a history of cardiovascular disease. They underwent cardiac magnetic resonance imaging (MRI) and spectroscopy (MRS). Cardiovascular risk factors, duration of HIV infection and cART and fasting plasma metabolite levels were recorded for each subject. Myocardial fibrosis, cardiac function and myocardial lipid content were assessed by MRI and MRS.

## Results

Compared to age-matched controls, HIV-infected subjects had on average 54% higher myocardial lipids, together with a two-fold elevation in plasma triglycerides (both p < 0.01). Myocardial fibrosis, predominantly in the basal infero-lateral wall of the left ventricle, was observed in 78% of HIV-infected subjects compared to 13% of controls (p < 0.001), irrespective of duration of infection or cART exposure. Peak myocardial systolic and diastolic longitudinal strain were also lower in HIV-infected individuals compared to controls (9% and 27% respectively, both p < 0.01), and so was left ventricular ejection fraction (68 ± 1% vs. 72 ± 1%, p<0.05). The differences in myocardial steatosis and fibrosis remained even after adjusting for potential confounders.

## Conclusions

Comprehensive Cardiac Magnetic Resonance imaging and spectroscopy revealed cardiac steatosis, alterations in cardiac function and a high prevalence of myocardial fibrosis in a contemporary group of asymptomatic HIV-infected subjects. Cardiac steatosis and fibrosis may underlie cardiac dysfunction and increased cardiovascular morbidity and mortality in subjects living with HIV.

## Funding

Oxford NIHR Biomedical Research Centre programme. BHF Centre of Research. The British Heart Foundation (FS07/030) supported this work.

